# Interaction between smoking and diabetes in relation to subsequent risk of cardiovascular events

**DOI:** 10.1186/s12933-022-01447-2

**Published:** 2022-01-24

**Authors:** Yang Yang, Nianchun Peng, Gang Chen, Qin Wan, Li Yan, Guixia Wang, Yingfen Qin, Zuojie Luo, Xulei Tang, Yanan Huo, Ruying Hu, Zhen Ye, Guijun Qin, Zhengnan Gao, Qing Su, Yiming Mu, Jiajun Zhao, Lulu Chen, Tianshu Zeng, Xuefeng Yu, Qiang Li, Feixia Shen, Li Chen, Yinfei Zhang, Youmin Wang, Huacong Deng, Chao Liu, Shengli Wu, Tao Yang, Mian Li, Yu Xu, Min Xu, Zhiyun Zhao, Tiange Wang, Jieli Lu, Yufang Bi, Weiqing Wang, Guang Ning, Qiao Zhang, Lixin Shi

**Affiliations:** 1 Department of Endocrinology and Metabolism, Affiliated Hospital of Guizhou Medical University, Guizhou Medical University, Guiyang, China; 2 Fujian Provincial Hospital, Fujian Medical University, Fuzhou, China; 3 The Affiliated Hospital of Luzhou Medical College, Luzhou, China; 4 Sun Yat-Sen Memorial Hospital, Sun Yat-Sen University, Guangzhou, China; 5 The First Hospital of Jilin University, Changchun, China; 6 The First Affiliated Hospital of Guangxi Medical University, Nanning, China; 7 The First Hospital of Lanzhou University, Lanzhou, China; 8 Jiangxi Provincial People’s Hospital Affiliated To Nanchang University, Nanchang, China; 9 Zhejiang Provincial Center for Disease Control and Prevention, Hangzhou, China; 10 Division of Endocrinology, Department of Internal Medicine, The First Affiliated Hospital of Zhengzhou University, Zhengzhou, China; 11 Dalian Municipal Central Hospital, Dalian, China; 12 Xinhua Hospital Affiliated to Shanghai Jiaotong University School of Medicine, Shanghai, China; 13 Chinese People’s Liberation Army General Hospital, Beijing, China; 14 Shandong Provincial Hospital Affiliated To Shandong University, Jinan, China; 15 Union Hospital, Tongji Medical College, Huazhong University of Science and Technology, Wuhan, China; 16 Tongji Hospital, Tongji Medical College, Huazhong University of Science and Technology, Wuhan, China; 17 The Second Affiliated Hospital of Harbin Medical University, Harbin, China; 18 The First Affiliated Hospital of Wenzhou Medical University, Wenzhou, China; 19 Qilu Hospital of Shandong University, Jinan, China; 20 Central Hospital of Shanghai Jiading District, Shanghai, China; 21 The First Affiliated Hospital of Anhui Medical University, Hefei, China; 22 The First Affiliated Hospital of Chongqing Medical University, Chongqing, China; 23 Jiangsu Province Hospital On Integration of Chinese and Western Medicine, Nanjing, China; 24 Karamay Municipal People’s Hospital, Xinjiang, China; 25 The First Affiliated Hospital of Nanjing Medical University, Nanjing, China; 26 Department of Endocrine and Metabolic Diseases, Shanghai Institute of Endocrine and Metabolic Diseases, Ruijin Hospital, Shanghai Jiao Tong University School of Medicine, Shanghai, China; 27 Shanghai National Clinical Research Center for Metabolic Diseases, Key Laboratory for Endocrine and Metabolic Diseases of the National Health Commission of the PR China, Shanghai National Center for Translational Medicine, Ruijin Hospital, Shanghai Jiao Tong University School of Medicine, Shanghai, China; 28 Department of Endocrinology and Metabolism, GuiQian International General Hospital, Guiyang, China

**Keywords:** Diabetes, Risk of cardiovascular disease, Smoking, Risk factor management

## Abstract

**Background:**

Whether smoking modifies the associations of diabetes and risk factor management with subsequent risk of cardiovascular disease (CVD), and whether the smoking related CVD risk differs among people with and without diabetes are unclear. This study aimed to examine the associations and interactions of smoking, diabetes, and risk factor management in relation to incident CVD.

**Methods:**

This nationwide, population-based, prospective cohort study of 20 communities from various geographic regions recruited adults aged 40 years or older during 2011–2012. The follow-up survey was conducted between 2014 and 2016. This study included 126,181 participants who were free from CVD at baseline.

**Results:**

Study participants included 19,397 current smokers (15.4%), 6,049 former smokers (4.8%), and 100,735 never smokers (79.8%). Mean (SD) age ranged from 55.8 (8.6) years to 60.7 (9.1) years. Compared with never smokers, heavy smokers exhibited a greater risk of CVD events among participants with diabetes (multivariable-adjusted hazard ratio [HR], 1.45; 95% CI, 1.17–1.78) than among participants without diabetes (HR, 1.20; 95% CI, 1.01–1.42; P for interaction = 0.006). Compared with participants without diabetes, participants with diabetes who were never smokers and had 5 or more controlled risk factors showed no significantly excess CVD risk (HR, 0.93; 95% CI, 0.71–1.22), but the cardiovascular benefits from risk factor management were counteracted among participants with diabetes who were current smokers (HR, 1.28; 95% CI, 0.77–2.14) or former smokers (HR, 1.22; 95% CI, 0.66–2.28).

**Conclusions:**

Smoking and diabetes interacted with each other in relation to increased risk of CVD events, and the beneficial effect of risk factor management on CVD risk among participants with diabetes was attenuated by current or former smoking.

**Supplementary Information:**

The online version contains supplementary material available at 10.1186/s12933-022-01447-2.

## Introduction

Cigarette smoking is the leading preventable cause of morbidity and mortality worldwide, responsible for 11.5% (equivalent to 6.4 million) of global deaths annually [1, 2]. In China, smoking has been ranked as the most important lifestyle risk factor contributing to deaths and disability-adjusted life-years [3]. Smoking substantially increases the risk of cardiovascular disease (CVD) in both the general population and patients with type 2 diabetes [4–6]. Compared with people without diabetes, patients with diabetes have a twofold additional hazard of CVD, occurring earlier and with greater severity [7–9]; meanwhile CVD remains the most common cause of mortality among patients with diabetes [10]. Therefore, interventions to prevent or quit smoking, together with cardiovascular risk factor management such as glycemic control and the management of blood pressure and cholesterol, are key recommendations for the prevention of CVD in diabetes [11, 12].

Smoking prevention and cessation interventions compare favorably with the management of other cardiovascular risk factors, in terms of both health improvements and cost savings [13]. However, a considerable proportion of people are likely to continue smoking even after a diagnosis of diabetes [14], and the successful rate of smoking cessation interventions for smokers with diabetes is not ideal [15]. Evaluating the interplay between cigarette smoking and diabetes status in relation to CVD risk is of the essence for prioritizing precise clinical and public health strategies to reduce the burden of CVD among patients with diabetes. Thus far, whether current smoking or smoking cessation modifies the associations of diabetes and risk factor management with the incidence of CVD, and whether the smoking-related CVD risk differs among people with and without diabetes are still unknown.

To fill this knowledge gap, we investigated the bidirectional interactions of active smoking and smoking cessation with diabetes status on subsequent risk of CVD events, and examined the association of cardiovascular risk factor management in diabetes with the risk of CVD across smoking status categories in a nationwide prospective cohort study.

## Methods

### Study participants

The China Cardiometabolic Disease and Cancer Cohort (4C) Study is a multicenter, population-based, prospective cohort study [16, 17]. The baseline survey was conducted between 2011 and 2012, and 19,3846 men and women aged 40 years or older were recruited from local resident registration systems of 20 communities from various geographic regions in China to represent the general population. The follow-up survey was conducted between 2014 and 2016; all participants were invited to attend an in-person visit, and 170,240 participants (87.8%) were followed up. The analysis included 126,181 participants who had complete baseline data on cigarette smoking, diabetes status, and covariates; were free from CVD at baseline; and had complete ascertainment of CVD events at the follow-up visit. This study was approved by the Medical Ethics Committee of Ruijin Hospital, Shanghai Jiao Tong University. All study participants provided written informed consent.

### Data collection

Data collection was performed in local community clinics by trained study personnel according to standard protocols. Questionnaires comprising information on demographic characteristics, dietary and lifestyle factors, family history, and medical history were administered by trained interviewers. Education attainment was classified as less than high school (< 9 years) and high school or further education (≥ 9 years). A food frequency questionnaire was used to collect habitual dietary intake by asking the consumption frequency and portion size of typical food items during the previous 12 months. International Physical Activity Questionnaire was used to assess physical activity [18], and Metabolic equivalent (MET) was calculated to evaluate average weekly energy expenditure [19]. Physical activity was classified as active (≥ 600 MET-min/week), insufficiently active (> 0 to < 600 MET-min/week), and inactive (0 MET-min/week). Average alcohol consumption was calculated by multiplying amount of alcohol consumed per drinking day by frequency.

Height and body weight were measured according to a standard protocol, and body mass index (BMI) was calculated as body weight in kilograms divided by the square of height in meters.

Three measurements of systolic blood pressure (SBP) and diastolic blood pressure (DBP) were obtained by using an automated electronic device (OMRON Model HEM-752 FUZZY, Dalian, China) in a seated position after at least a 5-min quiet rest, and the 3 readings were averaged for analysis. Hypertension was defined as SBP ≥ 140 mmHg, or DBP ≥ 90 mmHg, or taking antihypertensive medication.

All participants underwent a 2-h, 75-g oral glucose tolerance test (OGTT) after an overnight fast of at least 10 h, and blood samples were collected at 0 and 2 h during the test. Fasting and 2-h plasma glucose concentrations were measured locally using a glucose oxidase or hexokinase method within 2 h after blood sample collection under a stringent quality control program. Finger capillary whole blood samples were collected by the Hemoglobin Capillary Collection System (Bio-Rad Laboratories, Hercules, CA, USA) and were shipped and stored at 2℃ to 8℃ until glycated hemoglobin A1c (HbA1c) was measured within 4 weeks after collection by high-performance liquid chromatography using the VARIANT II Hemoglobin Testing System (Bio-Rad Laboratories) at the central laboratory in the Shanghai Institute of Endocrine and Metabolic Diseases, which was certificated by the National Glycohemoglobin Standardization Program and the College of American Pathologists Laboratory Accreditation Program.

Serum low-density lipoprotein (LDL) cholesterol, high-density lipoprotein (HDL) cholesterol, triglycerides, total cholesterol, and creatinine were measured at the central laboratory using an auto-analyzer (ARCHITECT ci16200, Abbott Laboratories, Chicago, IL, USA). Dyslipidemia was defined as LDL ≥ 160 mg/dL (4.14 mmol/L), or HDL < 40 mg/dL (1.04 mmol/L), or triglycerides ≥ 200 mg/dL (2.26 mmol/L), or total cholesterol ≥ 240 mg/dL (6.22 mmol/L), or taking lipid-lowering medications [20].

Estimated glomerular filtration rate (eGFR) was calculated using the Chronic Kidney Disease Epidemiology Collaboration equation [21]. Chronic kidney disease (CKD) was defined as eGFR < 60 mL/min/1.73 m [2].

### Assessment of cigarette smoking, diabetes, and risk factor management

Information on cigarette smoking was collected by a standard questionnaire. Current smokers were defined as participants who reported smoking cigarettes every day or almost every day, with at least 7 cigarettes per week for at least 6 months [2]. Intensity of current smoking was classified into light (< 10 cigarettes per day), moderate (10- < 20 cigarettes per day), and heavy (≥ 20 cigarettes per day) levels by number of cigarettes smoked [22]. Former smokers were defined as participants who reported not currently smoke cigarettes but had smoked at least 7 cigarettes per week for at least 6 months in a lifetime [2]. Former smokers were classified into short-term quitters (quit for < 4 years), medium-term quitters (quit for 4- < 8 years), and long-term quitters (quit for ≥ 8 years) by years since smoking cessation. Never smokers were defined as participants who reported never smoked or had not smoked cigarettes regularly (< 100 cigarettes in a lifetime) [2]. Passive smoking was defined as exposure to 1 or more smokers during childhood or adulthood.

According to the American Diabetes Association 2010 criteria, diabetes was defined as: fasting plasma glucose ≥ 126 mg/dL (7.0 mmol/L), or OGTT 2-h plasma glucose ≥ 200 mg/dL (11.1 mmol/L), or HbA1c ≥ 6.5%, or a self-reported previous diagnosis of diabetes by health care professionals [23].

For participants with diabetes, we considered optimal management of 6 cardiovascular risk factors in accordance with previous studies: (1) fruits and vegetables intake ≥ 4.5 cup/day [24]; (2) physical activity ≥ 600 MET-min/week [24, 25]; (3) HbA1c < 6.5% [16, 26]; (4) SBP < 130 mmHg and DBP < 80 mmHg [27, 28]; (5) LDL cholesterol < 100 mg/dL [29]; (6) without CKD [29].

### Ascertainment of cardiovascular events

The outcome was the composite of incident fatal or nonfatal CVD events, including cardiovascular death, myocardial infarction, stroke, and hospitalized or treated heart failure. The detailed definitions of CVD events have been described previously [16]. Deaths and clinical outcomes were collected from local vital registries of the National Disease Surveillance Point System and National Health Insurance System. Two members of the outcome adjudication committee independently verified each clinical event and assigned potential causes of death, and discrepancies were adjudicated by discussions involving other members of the committee. The members of the outcome adjudication committee were unaware of the baseline clinical characteristics of each participant.

### Statistical analysis

Baseline characteristics of participants according to smoking status were summarized as means with standard deviations (SDs) for continuous variables and numbers with percentages for categorical variables. In the time-to-event analysis, participants were censored at the date of CVD diagnosis, death, or the end of follow-up, whichever occurred first. Person-time was calculated from the enrollment date to the censoring date for each participant. All participants had complete data of cigarette smoking, diabetes status, and covariates; missing data for risk factor variables were deleted from the analyses.

Cox proportional hazards models were used to calculate hazard ratios (HRs) and 95% confidence intervals (CIs) for CVD events associated with current smoking and smoking cessation among participants with and without diabetes, with adjustment for traditional CVD risk factors as well as baseline BMI, change in BMI during the follow-up period, and passive smoking. Associations of diabetes as well as elevated fasting glucose, elevated OGTT-2 h glucose, and elevated HbA1c with CVD events among current smokers, former smokers, and never smokers were also established. Multiplicative interactions between smoking and diabetes on CVD events were tested by including the product term (e.g., smoking status category × diabetes category) as well as smoking status and diabetes status in the models. We performed additional analyses to assess the associations and interactions between smoking and diabetes on non-fatal CVD events and CVD mortality. The numbers of well-managed risk factors were summed for each participant to represent an overall management of all 6 risk factors. Associations of individual and overall cardiovascular risk factor management with CVD events among participants with diabetes according to smoking status, as compared with participants without diabetes were analyzed.

Statistical significance was assessed using a two-sided P value of < 0.05. Statistical analyses used SAS software, version 9.4 (SAS Institute Inc).

## Results

Baseline characteristics of participants according to smoking status are shown in Table 1. Of the 126,181 participants, there were 19,397 current smokers (15.4%), 6,049 former smokers (4.8%), and 100,735 never smokers (79.8%). Compared with never smokers, current smokers or former smokers had higher proportions of men, were more likely to be exposed to passive smoking in childhood, and had lower proportions of family history of CVD. Current smokers consumed fewer fruits and vegetables, were less physically active, and had higher levels of alcohol consumption than former smokers or never smokers. Former smokers had poorer glucose profiles and higher blood pressures than current smokers or never smokers.

**Table 1:** Baseline characteristics of participants according to smoking status

Characteristic	Current smokers	Former smokers			Never smokers
		Quit for < 4 years	Quit for 4- < 8 years	Quit for ≥ 8 years	
Number of participants	19,397	2405	848	2796	100,735
Age, year	55.8 (8.6)	58.2 (8.9)	59.4 (8.5)	60.7 (9.1)	56.4 (9.0)
Men, n (%)	18,230 (94.0)	2247 (93.4)	800 (94.3)	2684 (96.0)	19,609 (19.5)
High school or further education, n (%)	6761 (34.9)	953 (39.6)	372 (43.9)	1237 (44.2)	36,787 (36.5)
Family history of diabetes, n (%)	2310 (11.9)	305 (12.7)	104 (12.3)	370 (13.2)	13,292 (13.2)
Family history of CVD, n (%)	2575 (13.3)	332 (13.8)	137 (16.2)	416 (14.9)	17,491 (17.4)
BMI, kg/m^2^	24.4 (3.5)	25.0 (3.5)	25.3 (3.5)	25.3 (3.6)	24.6 (3.6)
Passive smoking exposure in childhood, n (%)	11,708 (60.4)	1450 (60.3)	491 (57.9)	1642 (58.7)	45,019 (44.7)
Passive smoking exposure in adulthood, n (%)	7536 (38.9)	770 (32.0)	249 (29.4)	886 (31.7)	36,188 (35.9)
Fruits and vegetables intake, cup/day	3.9 (3.6)	4.2 (4.0)	4.7 (6.5)	5.2 (26.9)	4.2 (5.6)
Physical activity, n (%)					
Active	11,474 (59.2)	1609 (66.9)	607 (71.6)	2078 (74.3)	65,414 (64.9)
Insufficiently active	7425 (38.3)	734 (30.5)	224 (26.4)	671 (24.0)	32,590 (32.4)
Inactive	498 (2.6)	62 (2.6)	17 (2.0)	47 (1.7)	2731 (2.7)
Alcohol consumption, g/day	24.8 (45.1)	21.3 (44.6)	20.1 (40.7)	18.3 (36.2)	2.3 (13.7)
Glucose profile					
Fasting glucose, mg/dL	108.6 (32.4)	112.0 (33.2)	114.4 (33.0)	113.3 (34.2)	106.6 (28.6)
OGTT-2 h glucose, mg/dL	143.9 (73.9)	154.7 (76.5)	164.5 (77.3)	160.0 (78.6)	147.8 (66.8)
HbA1c, %	6.0 (1.1)	6.1 (1.2)	6.2 (1.2)	6.2 (1.1)	6.0 (1.0)
Blood pressure					
SBP, mm Hg	132.9 (20.0)	136.3 (20.3)	136.9 (18.7)	136.7 (20.0)	132.6 (20.9)
DBP, mm Hg	79.8 (11.5)	80.7 (11.3)	81.6 (11.2)	80.8 (11.2)	78.0 (11.0)
Lipid profile					
LDL cholesterol, mg/dL	108.1 (32.3)	108.4 (33.1)	105.7 (33.3)	108.5 (33.6)	112.2 (34.2)
HDL cholesterol, mg/dL	49.3 (15.0)	48.3 (14.1)	47.5 (14.7)	47.6 (13.1)	52.5 (13.8)
Triglycerides, mg/dL	158.6 (132.8)	154.4 (120.5)	157.9 (130.4)	148.5 (109.5)	142.0 (103.4)
Total cholesterol, mg/dL	187.3 (42.2)	186.4 (44.0)	182.6 (43.4)	185.0 (44.1)	193.5 (44.2)
eGFR, mL/min/1.73m^2^	95.6 (13.2)	92.7 (14.3)	91.1 (14.9)	89.8 (15.2)	93.6 (13.4)

Values are mean (SD) for continuous variables and number (proportion) for categorical variables. Overall, the numbers of missing values are 120 for fasting glucose, 683 for OGTT-2 h glucose, 179 for HbA1c, 666 for SBP, 673 for DBP, 276 for LDL cholesterol, 278 for HDL cholesterol, 380 for triglycerides, 259 for total cholesterol, and 297 for eGFR. Percentages may not sum to 100% due to rounding

**Fig. 1:**
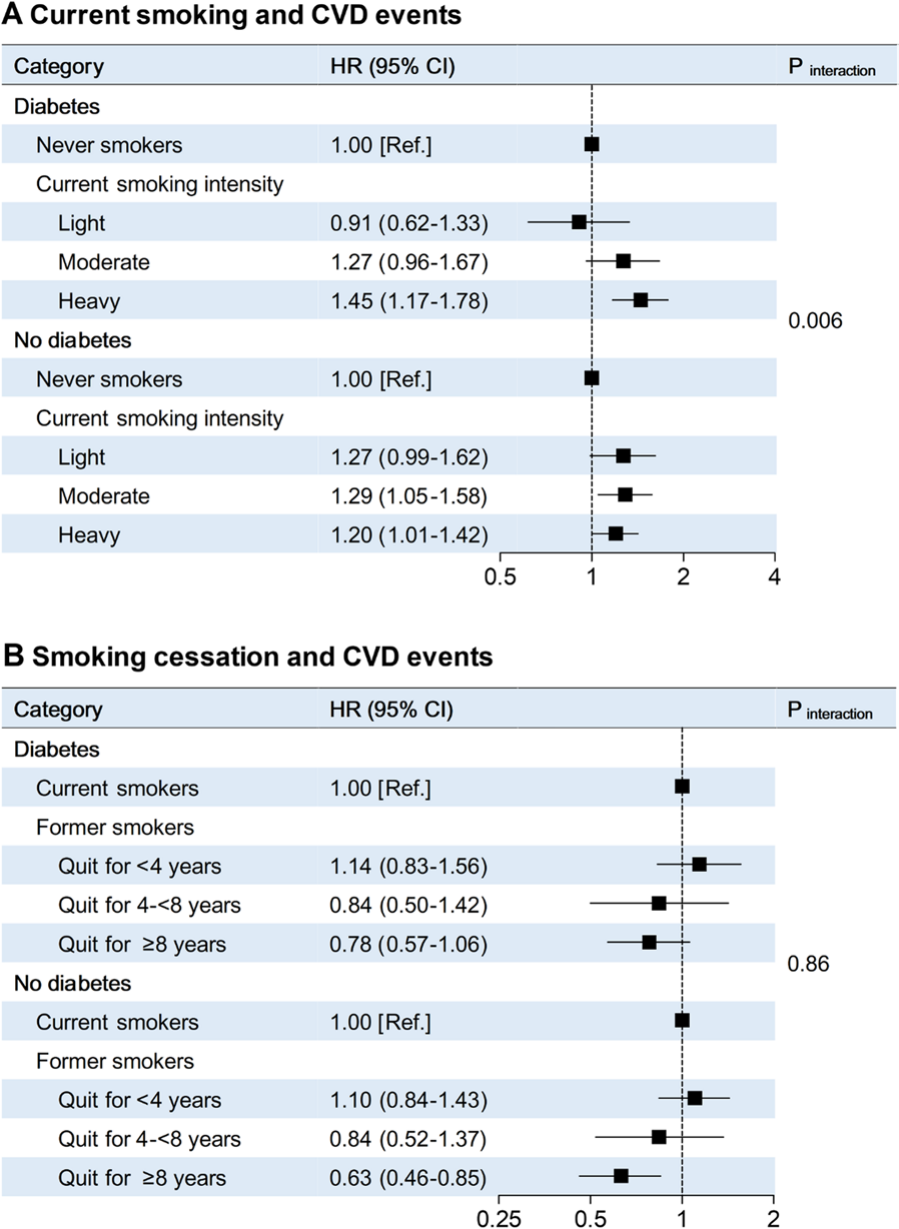
Association of current smoking and smoking cessation with CVD events among participants with and without diabetes. A. Number of participants included in the analysis: 19,397 current smokers and 100,735 never smokers. Light smokers (n = 3020) referred to participants who smoked < 10 cigarettes per day, moderate smokers (n = 5051) referred to participants who smoked 10- < 20 cigarettes per day, and heavy smokers (n = 11,326) referred to participants who smoked ≥ 20 cigarettes per day. HRs (95% CIs) and P value were adjusted for age, sex, education attainment (less than high school, high school or further education), family history of diabetes (yes, no), family history of CVD (yes, no), fruits and vegetables intake (< 4.5 cup/day, ≥ 4.5 cup/day), physical activity (active, insufficiently active, inactive), alcohol consumption, hypertension (yes, no), dyslipidemia (yes, no), baseline BMI, BMI change during follow-up, and passive smoking exposure in childhood and adulthood. B. Number of participants included in the analysis: 6,049 former smokers and 19,397 current smokers. HRs (95% CIs) and P value were adjusted for the same set of variables as shown in Panel A.

During 455,726 person-years (median, 3.2 years; interquartile range, 3.0–4.7 years) of follow-up, we documented 3,422 CVD events: 679 participants died from cardiovascular cause, 514 had myocardial infarction, 2,055 had stroke, and 251 had heart failure; participants may have experienced more than 1 CVD event. Compared with never smokers, current smokers had significantly higher hazard of CVD events among participants with diabetes (HR, 1.29; 95% CI, 1.08–1.54) than among participants without diabetes (HR, 1.24; 95% CI, 1.09–1.43; P for interaction = 0.034; Table 2). A significant interaction between current smoking intensity and diabetes on CVD events was also identified: the HRs for comparison of heavy smokers with never smokers were 1.45 (95% CI, 1.17–1.78) among participants with diabetes and 1.20 (95% CI, 1.01–1.42) among participants without diabetes (P for interaction = 0.006; Fig. 1A). A graded inverse association between years since smoking cessation and CVD events was observed: the HRs as compared with current smokers were 1.14 (95% CI, 0.83–1.56) for short-term quitters and 0.78 (95% CI, 0.57–1.06) for long-term quitters among participants with diabetes, and were 1.10 (95% CI, 0.84–1.43) for short-term quitters and 0.63 (95% CI, 0.46–0.85) for long-term quitters among participants without diabetes (P for interaction = 0.86; Fig. 1B). We observed similar associations of current smoking and smoking cessation with fatal and non-fatal CVD events, separately (Additional file 1: Figures S1, S2).

**Table 2:** Association of smoking with CVD events among participants with and without diabetes

Category	No. of participants	Person-years	Cases	HR (95% CI)			P for interaction^c^
				Adjusted for CVD risk factors^a^	Further adjusted for baseline BMI, and BMI change	Further adjusted for passive smoking^b^	
Diabetes							0.034
Never smokers	22,764	81,515	943	1.00 [Ref.]	1.00 [Ref.]	1.00 [Ref.]	
Former smokers	1913	6889	112	1.15 (0.92–1.43)	1.15 (0.92–1.43)	1.15 (0.93–1.43)	
Current smokers	4523	16,226	233	1.29 (1.08–1.54)	1.29 (1.08–1.53)	1.29 (1.08–1.54)	
No diabetes							
Never smokers	77,971	281,612	1604	1.00 [Ref.]	1.00 [Ref.]	1.00 [Ref.]	
Former smokers	4136	15,002	128	1.07 (0.88–1.30)	1.06 (0.88–1.30)	1.07 (0.88–1.30)	
Current smokers	14,874	54,483	402	1.23 (1.07–1.41)	1.24 (1.08–1.42)	1.24 (1.09–1.43)	

126,181 participants were included in the analysis

^a^Adjusted for age, sex, education attainment (less than high school, high school or further education), family history of diabetes (yes, no), family history of CVD (yes, no), fruits and vegetables intake (< 4.5 cup/day, ≥ 4.5 cup/day), physical activity (active, insufficiently active, inactive), alcohol consumption, hypertension (yes, no), and dyslipidemia (yes, no)

^b^Adjusted for CVD risk factors as listed above, as well as baseline BMI, BMI change during follow-up, and passive smoking exposure in childhood and adulthood

^c^Interaction of diabetes with smoking status in relation to CVD events was adjusted for CVD risk factors as listed above, as well as baseline BMI, BMI change during follow-up, and passive smoking exposure in childhood and adulthood

Diabetes as well as elevated fasting glucose, elevated OGTT-2 h glucose, and elevated HbA1c were associated with increased hazards of CVD events, and these associations were more prominent among current smokers and former smokers than among never smokers (Table 3). The multivariable-adjusted HRs for CVD events associated with diabetes were 1.61 (95% CI, 1.36–1.91) among current smokers, 1.70 (95% CI, 1.31–2.22) among former smokers, and 1.37 (95% CI, 1.26–1.49) among never smokers (P for interaction = 0.034). Significant interaction was also observed for elevated HbA1c (P for interaction = 0.011), whereas no statistically significant interaction was observed for elevated fasting glucose or elevated OGTT-2 h glucose. Similar but weaker results were observed for fatal and non-fatal CVD events separately (Additional file 1: Table S1).

**Table 3:** Association of diabetes, elevated fasting glucose, elevated OGTT-2 h glucose, and elevated HbA1c with CVD events among current, former, and never smokers

Category	No. of participants	Person-years	Cases	HR (95% CI)			P for interaction^c^
				Adjusted for CVD risk factors^a^	Further adjusted for baseline BMI, and BMI change	Further adjusted for passive smoking^b^	
Diabetes							0.034
Current smokers							
No diabetes	14,874	54,483	402	1.00 [Ref.]	1.00 [Ref.]	1.00 [Ref.]	
Diabetes	4523	16,226	233	1.60 (1.35–1.89)	1.61 (1.36–1.91)	1.61 (1.36–1.91)	
Former smokers							
No diabetes	4136	15,002	128	1.00 [Ref.]	1.00 [Ref.]	1.00 [Ref.]	
Diabetes	1913	6889	112	1.67 (1.28–2.16)	1.71 (1.31–2.22)	1.70 (1.31–2.22)	
Never smokers							
No diabetes	77,971	281,612	1604	1.00 [Ref.]	1.00 [Ref.]	1.00 [Ref.]	
Diabetes	22,764	81,515	943	1.38 (1.27–1.50)	1.37 (1.26–1.49)	1.37 (1.26–1.49)	
Elevated fasting glucose							0.11
Current smokers							
Fasting glucose < 126 mg/dL	16,722	61,110	484	1.00 [Ref.]	1.00 [Ref.]	1.00 [Ref.]	
Fasting glucose ≥ 126 mg/dL	2653	9522	147	1.69 (1.40–2.05)	1.71 (1.41–2.07)	1.71 (1.41–2.07)	
Former smokers							
Fasting glucose < 126 mg/dL	4931	17,868	172	1.00 [Ref.]	1.00 [Ref.]	1.00 [Ref.]	
Fasting glucose ≥ 126 mg/dL	1110	3997	68	1.61 (1.21–2.15)	1.66 (1.24–2.22)	1.66 (1.24–2.22)	
Never smokers							
Fasting glucose < 126 mg/dL	89,021	321,598	2011	1.00 [Ref.]	1.00 [Ref.]	1.00 [Ref.]	
Fasting glucose ≥ 126 mg/dL	11,624	41,208	532	1.47 (1.34–1.63)	1.46 (1.33–1.61)	1.46 (1.33–1.61)	
Elevated OGTT-2 h glucose							0.28
Current smokers							
OGTT-2 h glucose < 200 mg/dL	16,273	59,462	464	1.00 [Ref.]	1.00 [Ref.]	1.00 [Ref.]	
OGTT-2 h glucose ≥ 200 mg/dL	3035	10,918	163	1.56 (1.30–1.88)	1.57 (1.31–1.90)	1.57 (1.31–1.90)	
Former smokers							
OGTT-2 h glucose < 200 mg/dL	4697	16,999	160	1.00 [Ref.]	1.00 [Ref.]	1.00 [Ref.]	
OGTT-2 h glucose ≥ 200 mg/dL	1282	4646	75	1.44 (1.09–1.91)	1.45 (1.10–1.93)	1.45 (1.10–1.93)	
Never smokers							
OGTT-2 h glucose < 200 mg/dL	85,342	307,942	1844	1.00 [Ref.]	1.00 [Ref.]	1.00 [Ref.]	
OGTT-2 h glucose ≥ 200 mg/dL	14,869	53,371	684	1.44 (1.32–1.58)	1.43 (1.31–1.57)	1.44 (1.31–1.58)	
Elevated HbA1c							0.011
Current smokers							
HbA1c < 6.5%	16,402	59,910	467	1.00 [Ref.]	1.00 [Ref.]	1.00 [Ref.]	
HbA1c ≥ 6.5%	2955	10,652	167	1.69 (1.41–2.04)	1.72 (1.42–2.07)	1.72 (1.43–2.07)	
Former smokers							
HbA1c < 6.5%	4800	17,404	159	1.00 [Ref.]	1.00 [Ref.]	1.00 [Ref.]	
HbA1c ≥ 6.5%	1237	4446	78	1.77 (1.34–2.34)	1.83 (1.38–2.43)	1.83 (1.38–2.43)	
Never smokers							
HbA1c < 6.5%	85,759	309,451	1899	1.00 [Ref.]	1.00 [Ref.]	1.00 [Ref.]	
HbA1c ≥ 6.5%	14,849	53,219	643	1.39 (1.27–1.52)	1.38 (1.25–1.51)	1.37 (1.25–1.51)	

126,181 participants were included in the analysis. The numbers of missing values are 120 for fasting glucose, 683 for OGTT-2 h glucose, and 179 for HbA1c

^a^Adjusted for age, sex, education attainment (less than high school, high school or further education), family history of diabetes (yes, no), family history of CVD (yes, no), fruits and vegetables intake (< 4.5 cup/day, ≥ 4.5 cup/day), physical activity (active, insufficiently active, inactive), alcohol consumption, hypertension (yes, no), and dyslipidemia (yes, no)

^b^Adjusted for CVD risk factors as listed above, as well as baseline BMI, BMI change during follow-up, and passive smoking exposure in childhood and adulthood

^c^Interactions of diabetes, elevated fasting glucose, elevated OGTT-2 h glucose, and elevated HbA1c with smoking status in relation to CVD events were adjusted for CVD risk factors as listed above, as well as baseline BMI, BMI change during follow-up, and passive smoking exposure in childhood and adulthood

**Fig. 2:**
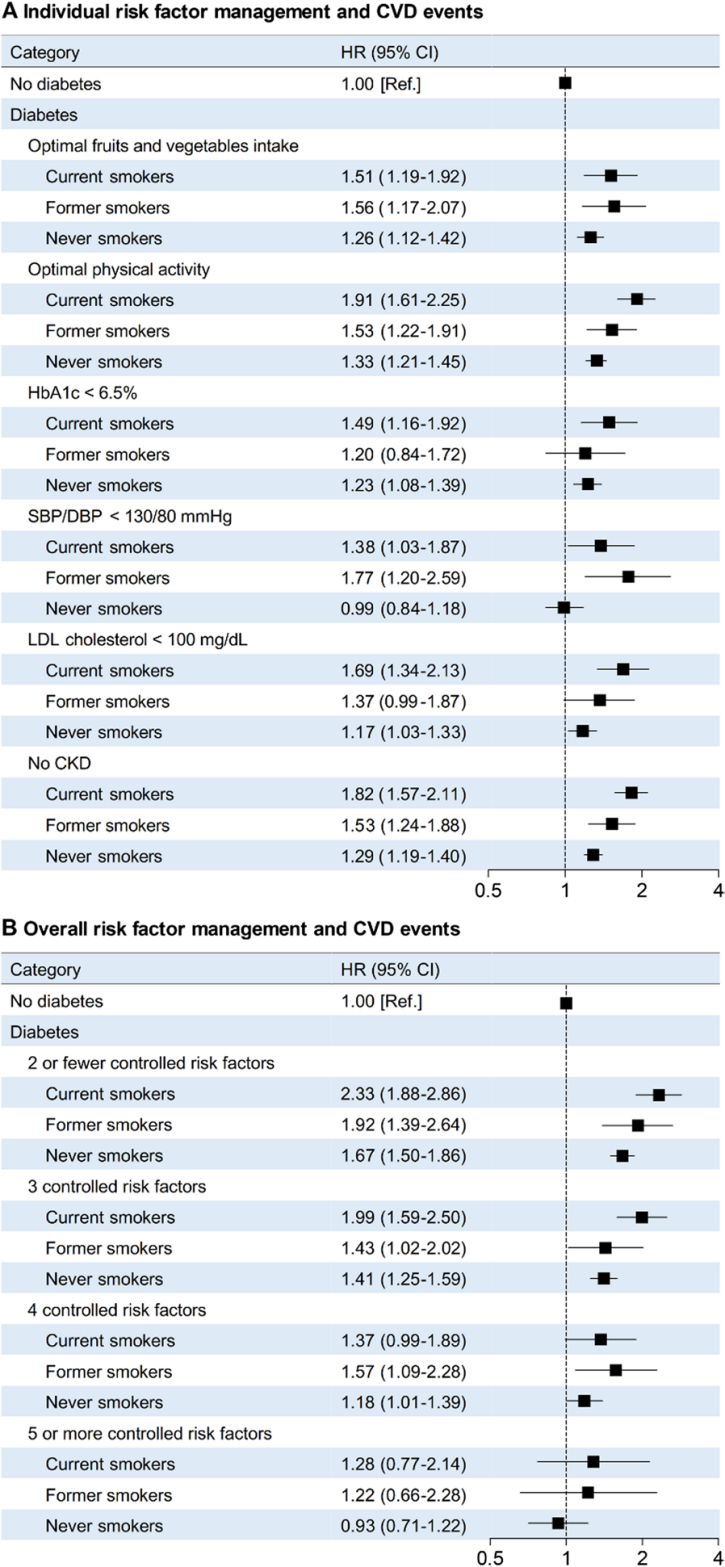
Association of cardiovascular risk factor management with CVD events among participants with diabetes according to smoking status, as compared with participants without diabetes. A. Number of participants included in the analysis: 96,981 without diabetes, 12,408 with diabetes and fruits and vegetables intake ≥ 4.5 cup/day, 19,921 with diabetes and physical activity ≥ 600 MET-min/week; 9980 with diabetes and HbA1c < 6.5%, 8052 with diabetes and SBP/DBP < 130/80 mmHg, 10,312 with diabetes and LDL cholesterol < 100 mg/dL, and 28,032 with diabetes and no CKD. HRs (95% CIs) were adjusted for age, sex, education attainment (less than high school, high school or further education), family history of diabetes (yes, no), family history of CVD (yes, no), fruits and vegetables intake (< 4.5 cup/day, ≥ 4.5 cup/day; not for fruits and vegetables intake analysis), physical activity (active, insufficiently active, inactive; not for physical activity analysis), alcohol consumption, hypertension (yes, no; not for SBP/DBP analysis), dyslipidemia (yes, no; not for LDL cholesterol analysis), baseline BMI, BMI change during follow-up, and passive smoking exposure in childhood and adulthood. B. Number of participants included in the analysis: 96,981 without diabetes, 9753 with diabetes and ≤ 2 controlled risk factors, 9667 with diabetes and 3 controlled risk factors, 6,846 with diabetes and 4 controlled risk factors, and 2934 with diabetes and ≥ 5 controlled risk factors. HRs (95% CIs) were adjusted for age, sex, education attainment (less than high school, high school or further education), family history of diabetes (yes, no), family history of CVD (yes, no), alcohol consumption, baseline BMI, BMI change during follow-up, and passive smoking exposure in childhood and adulthood.

Compared with participants without diabetes, participants with diabetes who had each individual controlled risk factor showed relatively lower hazards of CVD events among never smokers than among current or former smokers (Fig. 2A). Compared with participants without diabetes, participants with diabetes who had 5 or more controlled risk factors exhibited no significantly excess risk for CVD events (HR, 1.02; 95% CI, 0.81–1.27; Table 4). Such favorable effect of 5 or more controlled risk factors on CVD risk was more prominent among never smokers (HR, 0.93; 95% CI, 0.71–1.22), but was offset among current smokers (HR, 1.28; 95% CI, 0.77–2.14) or former smokers (HR, 1.22; 95% CI, 0.66–2.28; Fig. 2B).

**Table 4:** Association of cardiovascular risk factor management with CVD events among participants with diabetes, as compared with participants without diabetes

Category	Person-years	Cases	HR (95% CI)^a^
No diabetes	351,096	2134	1.00 [Ref.]
Diabetes			
Individual cardiovascular risk factor management			
Optimal fruits and vegetables intake	44,791	485	1.33 (1.20–1.47)
Optimal physical activity	71,765	898	1.43 (1.32–1.55)
HbA1c < 6.5%	35,669	391	1.27 (1.14–1.42)
SBP/DBP < 130/80 mmHg	29,104	221	1.12 (0.97–1.28)
LDL cholesterol < 100 mg/dL	37,126	397	1.28 (1.15–1.43)
No CKD	100,376	1154	1.39 (1.29–1.50)
Overall cardiovascular risk factor management			
2 or fewer controlled risk factors	34,699	542	1.79 (1.62–1.97)
3 controlled risk factors	34,582	428	1.50 (1.35–1.67)
4 controlled risk factors	24,629	238	1.25 (1.09–1.43)
5 or more controlled risk factors	10,720	80	1.02 (0.81–1.27)

Number of participants included in the analysis of individual risk factor management: 96,981 without diabetes, 12,408 with diabetes and fruits and vegetables intake ≥ 4.5 cup/day, 19,921 with diabetes and physical activity ≥ 600 MET-min/week; 9,980 with diabetes and HbA1c < 6.5%, 8,052 with diabetes and SBP/DBP < 130/80 mmHg, 10,312 with diabetes and LDL cholesterol < 100 mg/dL, and 28,032 with diabetes and no CKD. Number of participants included in the analysis of overall risk factor management: 96,981 without diabetes, 9,753 with diabetes and ≤ 2 controlled risk factors, 9,667 with diabetes and 3 controlled risk factors, 6,846 with diabetes and 4 controlled risk factors, and 2,934 with diabetes and ≥ 5 controlled risk factors

^a^For analysis of individual risk factor management: adjusted for age, sex, education attainment (less than high school, high school or further education), family history of diabetes (yes, no), family history of CVD (yes, no), fruits and vegetables intake (< 4.5 cup/day, ≥ 4.5 cup/day; not for fruits and vegetables intake analysis), physical activity (active, insufficiently active, inactive; not for physical activity analysis), alcohol consumption, hypertension (yes, no; not for SBP/DBP analysis), dyslipidemia (yes, no; not for LDL cholesterol analysis), baseline BMI, BMI change during follow-up, active smoking (never smoker, former smoker, current smoker), and passive smoking exposure in childhood and adulthood. For analysis of overall risk factor management: adjusted for age, sex, education attainment (less than high school, high school or further education), family history of diabetes (yes, no), family history of CVD (yes, no), alcohol consumption, baseline BMI, BMI change during follow-up, active smoking (never, former, current smoking), and passive smoking exposure in childhood and adulthood

## Discussion

In this nationwide prospective cohort study in China, we have provided novel and quantitative data about the interplay of cigarette smoking with diabetes and cardiovascular risk factor management on subsequent risk of CVD events. Compared with adults without diabetes, adults with diabetes were more susceptible to the deleterious effect of current smoking, especially heavy smoking, on CVD events. From another perspective, the excess CVD risks associated with diabetes and elevated glycemic traits were exacerbated among current smokers and former smokers, and the beneficial effect of risk factor management on CVD events among adults with diabetes seemed to be substantially attenuated by current or former smoking.

Our findings support a bidirectional interaction between smoking and diabetes on incident CVD events. Cigarette smoking and diabetes contribute independently to increased risk of CVD [4–8], and among people with diabetes, smoking is the key lifestyle risk factor for CVD [6]. In addition, current smokers have longer duration of exposure to passive smoking than never smokers and passive smoking is associated with an increased risk of CVD [30–32]. Given the considerable prevalence of smoking and the relatively low smoking cessation rate among people with diabetes and the effects of passive smoking on aspects of human health [14, 15], the specific effect that smoking poses to the diabetes population deserves special attention, and exposure to secondhand smoke should be minimized or avoided in order to reduce public health hazards [6, 33]. Prospective studies investigating the interaction between smoking and diabetes on CVD events are scarce and have revealed no evidence that the smoking-related CVD risk is greater in people with diabetes than in those without diabetes [34, 35]. In this study, taking advantage of a large nationwide sample with detailed records of smoking behaviors and comprehensive glycemic measurements, we extended previous evidence by showing that smoking and diabetes could amplify the deleterious effect of each other on subsequent risk of CVD, and the interaction between smoking and diabetes was quite consistent when diagnosing diabetes by each of the glycemic traits, especially elevated HbA1c. Our findings thus underline the importance of avoiding or quitting smoking for the effective prevention of CVD events, particularly for people with diabetes.

Our results also support a noteworthy conclusion that the favorable effects of optimal management of cardiovascular risk factors on incident CVD events among adults with diabetes could be considerably counteracted by smoking. Previous studies have demonstrated that substantial additional reductions in major CVD events in adults with diabetes can be achieved through ideal control of cardiovascular risk factors [16, 36–38]. In this study, the favorable cardiovascular effect of an ideal control of 5 or more risk factors in diabetes was only seen in never smokers; the CVD risk remained high among adults with diabetes who were current smokers or former smokers, even with optimal risk factor management. These results suggest that smoking is an important barrier for people with diabetes to gain cardiovascular benefits through risk factor management.

The precise mechanisms to explain our observations are unclear. Theoretically, both smoking and diabetes contribute to the development of cardiac dysfunction via promoting oxidative stress, inducing endothelial injury, and leading formation of atheroma [39], and these shared pathogenic pathways may underlie the interplay between smoking and diabetes on CVD. In addition, smoking is independently associated with the devastating cardiovascular health consequences for people with diabetes [39, 40]. Therefore, the benefits of ideal management of other risk factors may not fully outweigh the smoking-related cardiovascular risk. Experimental studies are needed to shed light on potential mechanisms responsible for the interplay between smoking and diabetes on CVD risk.

The strengths of this study included the large nationwide sample size, the prospective study design, the detailed records of smoking behaviors and comprehensive measurements of glycemic traits, and the well-validated definitions of the CVD outcomes. This study also has several limitations. First, the self-reported nature of smoking information may underestimate the prevalence of smoking as ascertained by cotinine measurement, and biochemical verification is needed to confirm the findings of this study. Second, we could not fully consider the residual and unmeasured confounders, such as the use of aspirin and metformin, which has been suggested to help prevent CVD risk in certain patients [41]. Third, the large sample size of this study can enhance the reliability of our findings and has advantages in permitting stratified analyses. However, because of the large sample size in this study, a statistically significant finding might not necessarily be clinically significant; thus, significant findings derived from this study must be analyzed and interpreted with caution. Fourth, similar to previous National Smoking Survey [42] and China Noncommunicable Disease Surveillance [43], the proportion of smokers were extremely low in Chinese women. Therefore, the limited number of smokers restricted the statistical power to perform sex-stratification analysis in this study. Fifth, the participants were middle-aged and elderly Chinese adults, and a majority of current smokers and former smokers were men. Thus, the generalizability of our findings to other groups should be cautious.

## Conclusions

Smoking and diabetes significantly interacted with each other in imposing increased risks of subsequent CVD events, and the cardiovascular benefits of risk factor management in diabetes could be counteracted by former or current smoking. Our findings highlight the particular importance of avoiding or quitting smoking to reduce the preventable burden of CVD in people with diabetes.

## Supplementary Information

**Additional file 1: Table S1**. Association of diabetes, elevated fasting glucose, elevated OGTT-2h glucose, and elevated HbA1c with non-fatal CVD events and CVD mortality among current, former, and never smokers. 126,181 participants were included in the analysis. The numbers of missing values are 120 for fasting glucose, 683 for OGTT-2h glucose, and 179 for HbA1c. aAdjusted for age, sex, education attainment (less than high school, high school or further education), family history of diabetes (yes, no), family history of CVD (yes, no), fruits and vegetables intake (<4.5 cup/day, ≥4.5 cup/day), physical activity (active, insufficiently active, inactive), alcohol consumption, hypertension (yes, no), dyslipidemia (yes, no), baseline BMI, BMI change during follow-up, and passive smoking exposure in childhood and adulthood. bPerson-years for CVD mortality was longer than that for non-fatal CVD events, because participants may have experienced more than one CVD event. **Figure S1**. Association of current smoking with non-fatal CVD events and CVD mortality among participants with and without diabetes. Number of participants included in the analysis: 19,397 current smokers and 100,735 never smokers. Light smokers (n=3,020) referred to participants who smoked <10 cigarettes per day, moderate smokers (n=5,051) referred to participants who smoked 10-<20 cigarettes per day, and heavy smokers (n=11,326) referred to participants who smoked ≥20 cigarettes per day. HRs (95% CIs) and P value were adjusted for age, sex, education attainment (less than high school, high school or further education), family history of diabetes (yes, no), family history of CVD (yes, no), fruits and vegetables intake (<4.5 cup/day, ≥4.5 cup/day), physical activity (active, insufficiently active, inactive), alcohol consumption, hypertension (yes, no), dyslipidemia (yes, no), baseline BMI, BMI change during follow-up, and passive smoking exposure in childhood and adulthood. **Figure S2**. Association of smoking cessation with non-fatal CVD events and CVD mortality among participants with and without diabetes. Number of participants included in the analysis: 6,049 former smokers and 19,397 current smokers. HRs (95% CIs) and P value were adjusted for age, sex, education attainment (less than high school, high school or further education), family history of diabetes (yes, no), family history of CVD (yes, no), fruits and vegetables intake (<4.5 cup/day, ≥4.5 cup/day), physical activity (active, insufficiently active, inactive), alcohol consumption, hypertension (yes, no), dyslipidemia (yes, no), baseline BMI, BMI change during follow-up, and passive smoking exposure in childhood and adulthood.

## Data Availability

The datasets used and analyzed during the current study are available from the corresponding author on reasonable request.

## Abbreviations

CKD: Chronic kidney disease
CVD: Cardiovascular disease
DBP: Diastolic blood pressure
eGFR: Estimated glomerular filtration rate
HbA1c: Hemoglobin A1c
HDL: High-density lipoprotein
LDL: Low-density lipoprotein
MET: Metabolic equivalent
OGTT: Oral glucose tolerance test
SBP: Systolic blood pressure
