# Supplementary material for: Interaction between smoking and diabetes in relation to subsequent risk of cardiovascular events
Source: Cardiovasc Diabetol. 2022 Jan 24;21:14. doi: 10.1186/s12933-022-01447-2 (PMC8787903; doi:10.1186/s12933-022-01447-2)

**Additional file 1：**

**Table S1.** Association of diabetes, elevated fasting glucose, elevated OGTT-2h glucose, and elevated HbA1c with non-fatal CVD events and CVD mortality among current, former, and never smokers

| **Category** | **Non-fatal CVD events** | | | | **CVD mortality** | | | |
| --- | --- | --- | --- | --- | --- | --- | --- | --- |
|  | **Person-years** | **Cases** | **HR (95% CI) ^a^** | **P for interaction** | **Person-years ^b^** | **Cases** | **HR (95% CI) ^a^** | **P for interaction** |
| Diabetes |  |  |  | 0.18 |  |  |  | 0.070 |
| Current smokers |  |  |  |  |  |  |  |  |
| No diabetes | 54483 | 323 | 1.00 [Ref.] |  | 54972 | 79 | 1.00 [Ref.] |  |
| Diabetes | 16226 | 183 | 1.53 (1.27-1.85) |  | 16519 | 50 | 1.95 (1.34-2.83) |  |
| Former smokers |  |  |  |  |  |  |  |  |
| No diabetes | 15002 | 102 | 1.00 [Ref.] |  | 15158 | 26 | 1.00 [Ref.] |  |
| Diabetes | 6889 | 78 | 1.49 (1.10-2.03) |  | 6992 | 34 | 2.52 (1.47-4.30) |  |
| Never smokers |  |  |  |  |  |  |  |  |
| No diabetes | 281612 | 1313 | 1.00 [Ref.] |  | 283433 | 291 | 1.00 [Ref.] |  |
| Diabetes | 81515 | 744 | 1.36 (1.23-1.49) |  | 82638 | 199 | 1.47 (1.21-1.77) |  |
| Elevated fasting glucose |  |  |  | 0.53 |  |  |  | 0.028 |
| Current smokers |  |  |  |  |  |  |  |  |
| Fasting glucose <126 mg/dL | 61110 | 390 | 1.00 [Ref.] |  | 61703 | 94 | 1.00 [Ref.] |  |
| Fasting glucose ≥126 mg/dL | 9522 | 113 | 1.55 (1.25-1.93) |  | 9707 | 34 | 2.64 (1.74-4.00) |  |
| Former smokers |  |  |  |  |  |  |  |  |
| Fasting glucose <126 mg/dL | 17868 | 132 | 1.00 [Ref.] |  | 18061 | 40 | 1.00 [Ref.] |  |
| Fasting glucose ≥126 mg/dL | 3997 | 48 | 1.52 (1.08-2.13) |  | 4063 | 20 | 2.09 (1.18-3.70) |  |
| Never smokers |  |  |  |  |  |  |  |  |
| Fasting glucose <126 mg/dL | 321598 | 1630 | 1.00 [Ref.] |  | 323916 | 381 | 1.00 [Ref.] |  |
| Fasting glucose ≥126 mg/dL | 41208 | 423 | 1.46 (1.30-1.63) |  | 41831 | 109 | 1.53 (1.23-1.91) |  |
| Elevated OGTT-2h glucose |  |  |  | 0.48 |  |  |  | 0.36 |
| Current smokers |  |  |  |  |  |  |  |  |
| OGTT-2h glucose <200 mg/dL | 59462 | 370 | 1.00 [Ref.] |  | 60022 | 94 | 1.00 [Ref.] |  |
| OGTT-2h glucose ≥200 mg/dL | 10918 | 131 | 1.55 (1.26-1.91) |  | 11134 | 32 | 1.68 (1.10-2.57) |  |
| Former smokers |  |  |  |  |  |  |  |  |
| OGTT-2h glucose <200 mg/dL | 16999 | 124 | 1.00 [Ref.] |  | 17183 | 36 | 1.00 [Ref.] |  |
| OGTT-2h glucose ≥200 mg/dL | 4646 | 53 | 1.36 (0.98-1.89) |  | 4717 | 22 | 1.69 (0.97-2.93) |  |
| Never smokers |  |  |  |  |  |  |  |  |
| OGTT-2h glucose <200 mg/dL | 307942 | 1502 | 1.00 [Ref.] |  | 310038 | 342 | 1.00 [Ref.] |  |
| OGTT-2h glucose ≥200 mg/dL | 53371 | 541 | 1.43 (1.30-1.59) |  | 54205 | 143 | 1.45 (1.19-1.78) |  |
| Elevated HbA1c |  |  |  | 0.12 |  |  |  | 0.013 |
| Current smokers |  |  |  |  |  |  |  |  |
| HbA1c <6.5 % | 59910 | 375 | 1.00 [Ref.] |  | 60481 | 92 | 1.00 [Ref.] |  |
| HbA1c ≥6.5 % | 10652 | 131 | 1.61 (1.30-1.98) |  | 10863 | 36 | 2.27 (1.51-3.41) |  |
| Former smokers |  |  |  |  |  |  |  |  |
| HbA1c <6.5 % | 17404 | 123 | 1.00 [Ref.] |  | 17585 | 36 | 1.00 [Ref.] |  |
| HbA1c ≥6.5 % | 4446 | 54 | 1.62 (1.16-2.26) |  | 4522 | 24 | 2.59 (1.49-4.48) |  |
| Never smokers |  |  |  |  |  |  |  |  |
| HbA1c <6.5 % | 309451 | 1538 | 1.00 [Ref.] |  | 311650 | 361 | 1.00 [Ref.] |  |
| HbA1c ≥6.5 % | 53219 | 514 | 1.38 (1.24-1.53) |  | 53960 | 129 | 1.40 (1.13-1.73) |  |

^a^ Adjusted for age, sex, education attainment (less than high school, high school or further education), family history of diabetes (yes, no), family history of CVD (yes, no), fruits and vegetables intake (<4.5 cup/day, ≥4.5 cup/day), physical activity (active, insufficiently active, inactive), alcohol consumption, hypertension (yes, no), dyslipidemia (yes, no), baseline BMI, BMI change during follow-up, and passive smoking exposure in childhood and adulthood.

^b^ Person-years for CVD mortality was longer than that for non-fatal CVD events, because participants may have experienced more than one CVD event.

**Figure S1.** Association of current smoking with non-fatal CVD events and CVD mortality among participants with and without diabetes
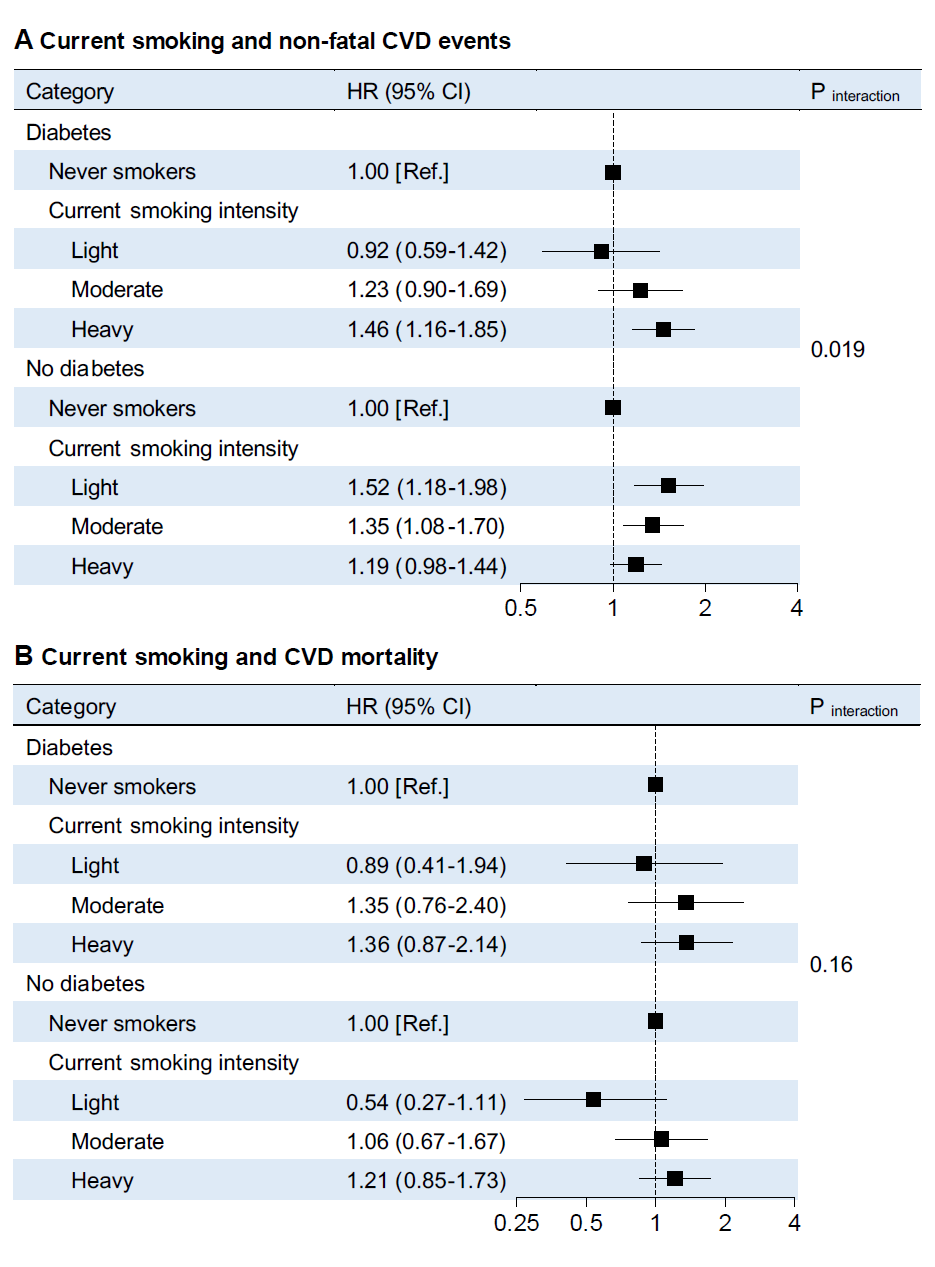


**Figure S2.** Association of smoking cessation with non-fatal CVD events and CVD mortality among participants with and without diabetes


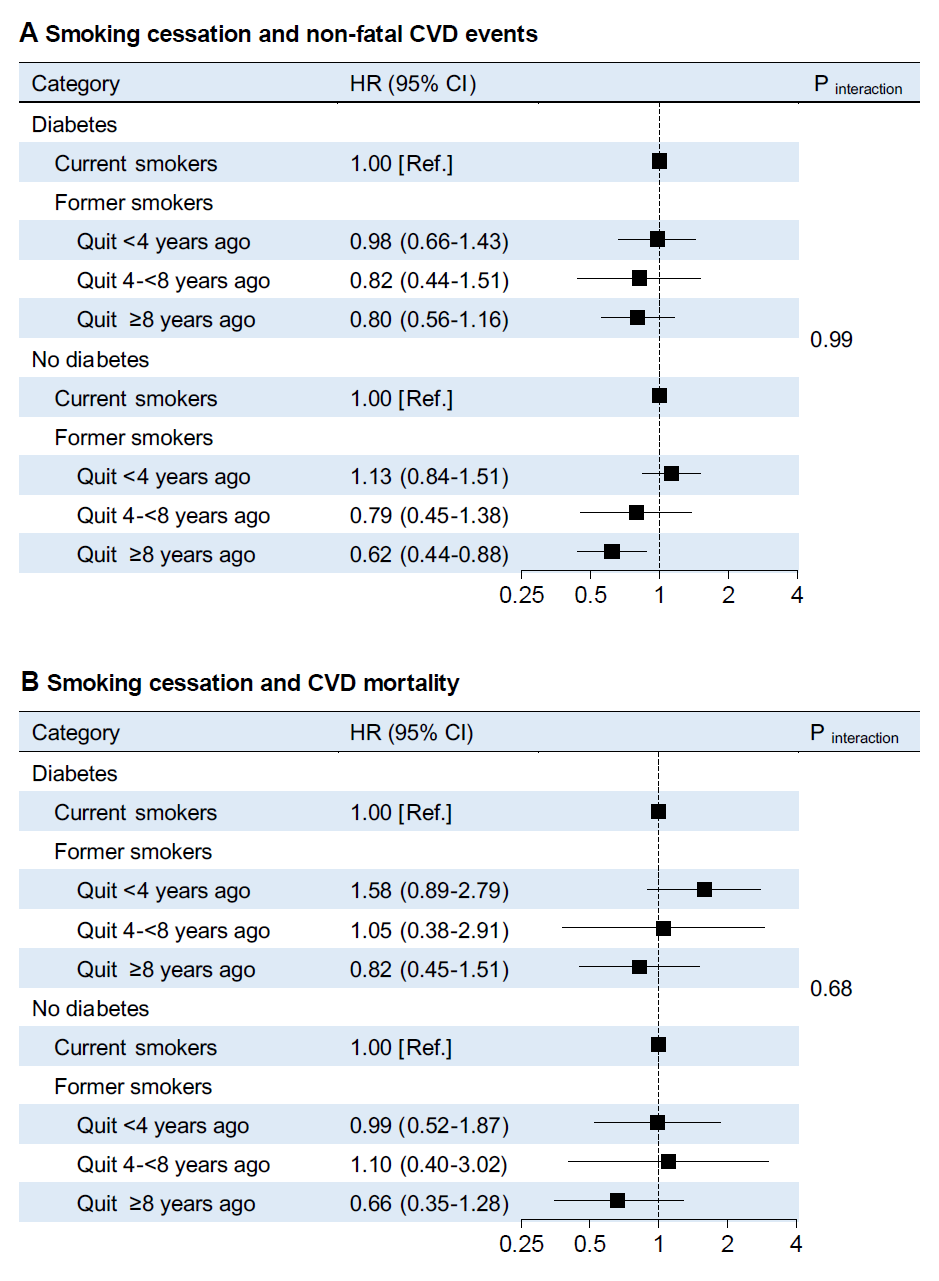

Supplement: Supplementary file 1 — Additional file 1: Table S1. Association of diabetes, elevated fasting glucose, elevated OGTT-2h glucose, and elevated HbA1c with non-fatal CVD events and CVD mortality among current, former, and never smokers. 126,181 participants were included in the analysis. The numbers of missing values are 120 for fasting glucose, 683 for OGTT-2h glucose, and 179 for HbA1c. aAdjusted for age, sex, education attainment (less than high school, high school or further education), family history of diabetes (yes, no), family history of CVD (yes, no), fruits and vegetables intake (<4.5 cup/day, ≥4.5 cup/day), physical activity (active, insufficiently active, inactive), alcohol consumption, hypertension (yes, no), dyslipidemia (yes, no), baseline BMI, BMI change during follow-up, and passive smoking exposure in childhood and adulthood. bPerson-years for CVD mortality was longer than that for non-fatal CVD events, because participants may have experienced more than one CVD event. Figure S1. Association of current smoking with non-fatal CVD events and CVD mortality among participants with and without diabetes. Number of participants included in the analysis: 19,397 current smokers and 100,735 never smokers. Light smokers (n=3,020) referred to participants who smoked <10 cigarettes per day, moderate smokers (n=5,051) referred to participants who smoked 10-<20 cigarettes per day, and heavy smokers (n=11,326) referred to participants who smoked ≥20 cigarettes per day. HRs (95% CIs) and P value were adjusted for age, sex, education attainment (less than high school, high school or further education), family history of diabetes (yes, no), family history of CVD (yes, no), fruits and vegetables intake (<4.5 cup/day, ≥4.5 cup/day), physical activity (active, insufficiently active, inactive), alcohol consumption, hypertension (yes, no), dyslipidemia (yes, no), baseline BMI, BMI change during follow-up, and passive smoking exposure in childhood and adulthood. Figure S2. Association of smoking ce [file 12933_2022_1447_MOESM1_ESM.docx]
